# Multi-kernel inception-enhanced vision transformer for plant leaf disease recognition

**DOI:** 10.1038/s41598-025-16142-x

**Published:** 2025-08-23

**Authors:** Sk Mahmudul Hassan, Kumar Sekhar Roy, Ruhul Amin Hazarika, Mehbub Alam, Mithun Mukherjee

**Affiliations:** 1 Manipal Institute of Technology Bengaluru, Manipal Academy of Higher Education, Manipal, Karnataka 576104 India; 2 Department of IT, Indian Institute of Information Technology, Guwahati, Assam 781015 India; 3 Nanjing University of Information Science and Technology, Nanjing, China

**Keywords:** Plant disease, Machine learning, Vision transformer, Deep learning, Plant sciences, Engineering

## Abstract

The timely and precise identification of diseases in plants is essential for efficient disease control and safeguarding of crops. Manual identification of diseases requires expert knowledge in the field, and finding people with domain knowledge is challenging. To overcome the challenge, computer vision-based machine learning techniques have been proposed by the researchers in recent years. Most of these solutions with the standard convolutional neural network (CNN) approaches use uniform background laboratory setup leaf images to identify the diseases. However, only a few works considered real-field images in their work. Therefore, there is a need for a robust CNN architecture that can identify the diseases in plants in both laboratory and real-field conditioned images. In this paper, we have proposed an Inception-Enhanced Vision Transformer (IEViT) architecture to identify diseases in plants. The proposed IEViT architecture extracts local as well as global features, which improves feature learning. The use of multiple filters with different kernel sizes efficiently uses computing resources to extract relevant features without the need for deeper networks. The robustness of the proposed architecture is established by hyper-parameter tuning and comparison with state-of-the-art. In the experiment, we consider five datasets with both laboratory-conditioned and real-field conditioned images. From the experimental results, we see that the proposed model outperforms state-of-the-art deep learning models with fewer parameters. The proposed model achieves an accuracy rate of 99.23% for the apple leaf dataset, 99.70% for the rice dataset, 97.02% for the ibean dataset, 76.51% for the cassava leaf dataset, and 99.41% for the plantvillage dataset.

## Introduction

The global demand for food production is met with some challenges in the form of plant disease, which makes a significant threat to production of crops^1^. Plant diseases are fueled by changing climatic conditions such as temperature. Hence, accurately and timely identifying these diseases is crucial to prevent their spread^2^. Traditional identification relies on visual inspection, which is labor-intensive, lacks precision, and is prone to human error^3^. Researchers have proposed several machine learning (ML)-based approaches to identify diseases in plants from the leaf images and broadly classify them into traditional ML-based approaches and deep learning (DL)-based approaches. The traditional ML-based approach includes algorithms such as K-nearest neighbour (KNN), support vector machine (SVM), random forest (RF), and decision tree (DT)^4^. The performance accuracy in this algorithm heavily depends on the extracted features from the images. Finding the set of features from the extracted features that gives optimum results is an important challenge in this approach.

CNNs have shown remarkable success in image analysis tasks, making them well-suited for the identification of visual symptoms associated with plant diseases. Their ability to automatically learn hierarchical features from images contributes to highly accurate disease classification. Several deep learning architectures such as, VGG16^5^, VGG19^5^, InceptionV3^6^, ResNet50^7^, MobileNet^8^, and EfficientNet^9^ are used in the identification of diseases. Despite their strengths, DL architectures fail to model global relationships effectively; deeper architectures widen receptive fields but risk losing low-level features in the process^10^. In recent times, deep learning with self-attention has been used in this field and gives prominent results. Despite the development of architecture and achieving high accuracy, it is still far from being implemented in real field conditions due to various reasons: (a) The number of parameters used in the state-of-the-art deep learning models is large and requires high computing devices to train the model. (b) Real-field images for the agricultural crops are unavailable. Designing a lightweight convolutional neural network (CNN) architecture that can effectively identify diseases in plants is an important area in research. In this view, Dosovitskiy et al.^11^ introduced the vision transformer (ViT) architecture in image processing and computer vision tasks. This architecture has achieved significant performance in classification with less memory and fewer parameters. Singh et al.^12^ used MobileViT architecture in the identification of plant leaf diseases with fewer parameter as compared to standard CNN models. However, a limited number of labelled images may often leads to class imbalance, which affects in models performances. A low-cost real time plant disease detection model named as PMVT, used by Li et al.^13^. They have replaced the convolution block with $7\times 7$ convolution and also integrated CBAM in standard ViT. In comparison with CNN, the ViT architecture heavily relies on model regularization and data augmentation while training on smaller datasets. The main reason is that the ViT architecture mainly focuses on extracting global features and long-distance features, whereas the CNN architecture focuses on local features. However, the combination of the CNN with the ViT architecture will enhance the feature extraction as the model will extract both local as well as global features. Further, hybridization of both CNN and ViT may increase the performance of the model. In this point of view, a novel lightweight Inception-Enhanced Vision Transformer (IEViT) architecture is proposed to identify the diseases in plants. The model combines features extracted from both Inception CNN and ViT architecture. This model begins with two inception blocks, where we use a parallel convolution filter to extract local features, followed by a stacked transformer block.

### Contribution and organization of the paper

The main contributions of the proposed architecture are as follows

- An Inception-Enhanced Vision Transformer (IEViT) architecture is proposed to identify the diseases in plants with wide and different conditioned images.
- To extract the local features, two inception blocks with parallel convolution are used, which use different filter sizes to extract the features.
- The model is lightweight and uses only 0.90M parameters, which is much less as compared to state-of-the-art deep learning models and can be feasible to implement in agriculture. The proposed model is implemented in different datasets, and the performances are compared with different state-of-the-art deep learning models. The proposed model outperforms several deep learning-based architectures.

The rest of the paper is organized as follows: Sect. “Related work” provides a brief discussion of several existing works. Section “Materials and methods” provides the details about the proposed models. Results and performance are discussed in Sect. “Experimental results and analysis”. Finally, the paper concludes in Sect. “Conclusion” with future scope.

## Related work

The performance of CNN in computer vision is impressive, and researchers have explored and designed several deep-learning models to identify diseases in plants. In this section, we have explored and summarized different deep-learning models used in plant disease detection. Mohanty et al.^14^ used two different deep learning architectures, AlexNet and GoogleNet, to identify the diseases in plants. In this paper, the authors used a large-scale plant dataset consisting of 54306 images of 38 different categories. Three different types of images were used, namely color, greyscale, and segmented images and recorded a maximum accuracy of 99.35%. Later, Ferentinos et al.^15^ used five different deep learning architectures to classify 58 distinct plant diseases and achieved an accuracy of 99.48% using VGG architecture.

Thakur et al.^16^ proposed a lightweight VGG-ICNN model for the identification of plant diseases in multiple plant disease datasets. In this paper, the authors used 4 convolution layers of VGG16 and three blocks of Inception v7. In their paper, the authors recorded an average accuracy rate of 99.16%. Later, a lightweight DenseNet (LWDN) proposed in^17^ to identify the diseases in plants achieved an accuracy rate of 99.36% in the plantvillage dataset^14^.

Too et al.^18^ suggested a fine-tuned deep learning architecture to identify different diseases in plants. Using DenseNet architecture^18^, they have recorded maximum accuracy. Further, Atila et al.^19^ used EfficientNet architecture to identify the diseases in plants, and they have compared the performances of the model with several state-of-art deep learning models and showed that EfficientNet architectures outperform other models. Moreover, Sangeetha et al.^20^ proposed an improved agro deep learning in the identification of panama wilts diseases in banana leaves. This technique used the arrangement of colour and shape changes in banana leaves to forecast the disease’s intensity and its effects and achieved an accuracy rate of 91.56%.

CNN, with self-attention, has also gained much attention and is widely used in the identification of plant diseases. Zeng et al.^21^ proposed a self-attention-based CNN (SACNN) model to identify different crop diseases. The SACNN model consists of the base network to extract global features and self-attention to extract the local features. In their work, different levels of noise were added in the images to evaluate the model performance and show SACNN outperform state-of-art deep learning models. Chen et al.^22^ proposed a lightweight attention-based deep learning architecture to identify the diseases in rice plants. The authors used the MobileNet-V2 pre-trained on ImageNet as the backbone network, and to improve the learning capability for minute lesion information, they incorporated an attention mechanism. The attention mechanism helps the network understand the significance of spatial points and inter-channel relationships for input features.

Moreover, Qian et al.^23^ proposed a deep CNN architecture to identify 4 different maize diseases. In this work, the authors divided the CNN architecture into three stages. Stage 1 extracts the image features and encodes them into a feature tokens matrix. Stage 2 is the core computation using multi-head self-attention, and finally, stage 3 is the classification stage. Deep attention dense CNN used by Pandey et al.^24^ to identify different plant diseases. Mixed sigmoid attention learning merging with basic dense learning used in this work as in dense learning features at higher layer considering all lower layer features that provide efficient training process. Further, attention learning strengthens the learning ability of the dense block. The authors achieved an accuracy rate of 97.55% on a real-time dataset consisting of 17 plant species. Later, Bhujel et al.^25^ proposed a lightweight self-attention CNN to identify different tomato leaf diseases. The model is proposed based on residual architecture and used 20 convolution layers, and after the 16th layer, they used an attention block.

Mohamed Zarboubi et al.^26^ proposed a CustomBottleneck-VGGNet to identify the different tomato leaf diseases. In this proposed approach author has used two layers of VGG16 followed by custom bottle neck layer with $1\times 1$ and $3\times 3$ convolutions . They have also included CBR (Convolution-Batch Norm-ReLU) and CBS (Convolution-BatchNorm-SiLU) layer. Author recorded an accuracy rate of 99.12% with 1.4M parameters.

Moreover, Ghost enlightened transformer (GET) architecture suggested by Lu et al.^27^ to identify grape diseases and pests. The performances of GeT suppress other deep learning models, achieve an accuracy rate of 98.14%, and are also faster and lighter. To enhance the feature extraction in ViT architecture, Yu et al.^28^ used inception convolution in ViT architecture to identify the diseases in plants. Four different datasets were used in this work, and the experimental results outperformed those of other deep learning models. Furthermore, A combination of CNN and ViT was used in the work^29^ to identify different diseases in plants. Three different datasets were used to evaluate the performances and show that fusion of attention with CNN blocks compensates the speed of the architecture. Mobile device compatible, PMVT a light weight transformer based architecture used in^13^ to identify the diseases in plant. In this paper, the author replaces the convolution block in MobileViT with an inverted residual structure and also incorporates CBAM into the ViT encoder. Multiple datasets were used to evaluate the performances of the model, and it achieved 1.6% higher performance than MobileNetV3 and 2.3% in Squeezenet.

Bellout et al.^30^ investigate 5 different YOLO model namely YOLOv5, YOLOX, YOLOv7, YOLOv8, and YOLO-NAS in identification of tomato leaf diseases. PlantDoc and PlantVillage dataset were used to investigate the result and achieved an accuracy of 93.1% using YOLOv5 model. A light weight IoT integrated DL based approach termed as LT-YOLOv10n, proposed by Abdelaaziz Bellout et al.^31^ to identify real-time tomato leaf disease detection. Author has incorporated CBAM and C3F layer in YOLOv10 architecture and developed a mobile-based application for the identification of diseases. The images from the public roboflow universe dataset, along with images from the PlantVillage dataset, were used to train the model and achieved an accuracy rate of 98.7%. Table 1 summarizes the articles discussed in the related section.

**Table 1:** Summarization of the existing work.

Paper references	DL model	Dataset	Class	Accuracy (%)
Mohanty et al.^14^	AlexNet, GoogleNet	PlantVillage^14^	38	99.34
Ferentinos et al.^15^	AlexNet, VGG, Overfeat, GoogleNet AlexNetOWTBn	PlantVillage^14^	58	99.48
Geetharamani et al.^32^	Nine layer CNN	PlantVillage^14^	38	96.46
Chen et al.^33^	VGGNet with two inception layer	Maize dataset^14^	4	84.25
Sethy et al.^34^	11 state-of-art CNN architecture with SVM for classification	Rice dataset^34^	4	98.38
Too et al.^18^	Fine tune 6 different CNN models	PlantVillage^14^	38	99.76
Atila et al.^19^	EfficientNet	PlantVillage^14^	38	99.38
Zeng et al.^21^	Self-attention CNN with Residual Connection	AES-CD9214 MK-D2 dataset^35^	6	95.59
Qian et al.^23^	Transformer and Multi- head attention	Maize dataset^14^	4	98.7
Pandey^24^	DADCNN-5	PlantVillage^14^	38	99.93
Bhujel et al.^25^	CNN with Multiple attention	Tomato leaf^14^	10	99.69
Lu et al.^27^	GET	GLDP12k dataset^27^	11	98.14
Yu et al.^28^	ViT architecture	Ibean^36^	3	99.22
Borhani et al.^29^	ViT architecture	Wheat rust^37^	3	100
Mohamed Zarboubi et al.^26^	CustomBottleneck- VGGNet	PlantVillage^14^	10	99.12
Abdelaaziz Bellout et al.^31^	LT-YOLOv10n	Roboflow Universe, PlantVillage^14^	9	98.7
Bellout et al.^30^	Multiple YOLO architecture	PlantVillage^14^ PlantDoc^38^	3	93.1

## Materials and methods

In this paper, we aim to design a lightweight deep learning architecture to identify diseases in plants and that can also be easily deployable in agriculture. In particular, we design a fusion of Inception block and ViT transformer architecture termed as IEViT to classify the diseases in plants.

### Inception block

Inception block in architecture first used in GoogleNet architecture by Szegedy et al.^6^. Increasing the layers in the DL architecture may result in overfitting in the model. In inception, it uses multiple filters of different sizes on the same layer. The outputs of all the convolution layers are concatenated together and forwarded to the next layer. The use of multiple filter sizes extracts better features, which increases the performance of the model. In this paper, we have used two inception blocks termed as InceptionA and InceptionB block. The normal convolution used in the inception block was replaced with depthwise separable convolution. The use of depthwise separable convolution reduces the number of parameters in the model. The parameter used in depthwise separable convolution is calculated as

1 $$\begin{aligned} S_{\text {depthwise}}= D_{K}^2\times M\times D_{F}^2+M\times N\times D_{K}^2\,. \end{aligned}$$

**Fig. 1:**
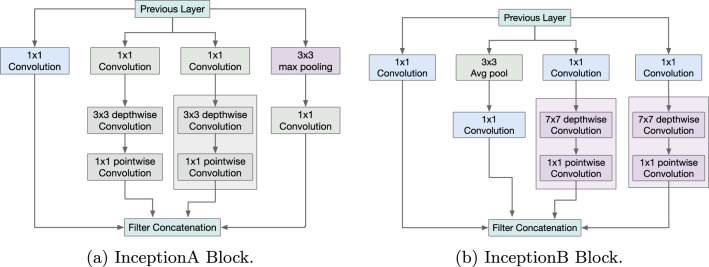
Block diagram of Inception Block used in Proposed architecture.

We write the computation cost in standard convolution as

2 $$\begin{aligned} \text {Cost}=D_{K}^2\times M\times N \times D_{F}^2\,, \end{aligned}$$

where $D_F$ is the input image dimension, $D_K$ is the kernel dimension, M is the number of channels and N is the number of kernel/filters. The inceptionA block consists of $1\times 1$ convolution, $1\times 1$ convolution followed by one $3\times 3$ depthwise separable convolution, $1\times 1$ convolution followed by two $3\times 3$ depthwise separable convolution and $3\times 3$ maxpooling followed by $1\times 1$ convolution as shown in figure. Similarly, in inceptionB block also we have used $3\times 3$ average pooling and $7\times 7$ depthwise separable convolution as shown in Fig. 1.

### Inverted bottleneck block (MV2)

**Fig. 2:**
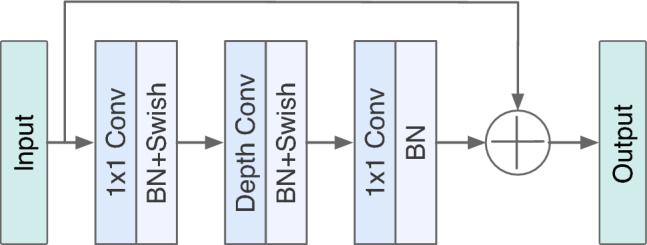
Block diagram of MV2 block.

The inverted residual structure was first used in MobileNet architecture^39^ and adopted in IEViT architecture, which undergoes feature enhancement and feature reduction in convolution. The block diagram of MV2 is shown in Fig. 2, and it is seen that MV2 uses three separate convolutions. At first, $1\times 1$ point-wise convolution is used to expand low-dimension to high-dimensional feature maps. Next, $3\times 3$ depth-wise separable convolution is used, followed by an activation function to achieve spatial filtering of the higher-dimensional data. Finally, $1\times 1$ point-wise convolution is used to project back to the low-dimensional subspace. The initial and final feature map is added using a residual connection.

We denote *X* as the input tensor to the inverted bottleneck block, $C_\text {in}$ represents the number of input channels, and $C_{out}$ represents the number of output channels. The first step involves expanding the number of channels by using a $1\times 1$ convolution followed by a non-linear activation function. Let *t* denote the expansion factor. The output of this layer is expressed as

3 $$\begin{aligned} X_\text {expanded} = \texttt {Swish}\big (\texttt {Conv2D}(X, C_\text {in}\times t, 1\times 1)\big ) \end{aligned}$$

Later, depthwise separable convolution is applied of kernel size $K\times K$ is applied with a depth multiplier $\alpha$ on each input channel. The output of this depthwise convolution layer is expressed as

4 $$\begin{aligned} X_{\text {depthwise}} = \texttt {DepthwiseConv2D}(X_{\texttt {ecpanded}},K\times K, depth\_\texttt {multiplier} = \alpha ) \end{aligned}$$

Following the depthwise convolution, a $1\times 1$ pointwise convolution is applied to combine information across channels to reduce the output channel to $C_{out}$. The output is expressed as

5 $$\begin{aligned} X_{out} = \texttt {Conv2D}(X_{\texttt {depthwise}},C_{\text {out}},1\times 1) \,. \end{aligned}$$

Finally, a residual connection is added between the input and output of the block. The output is expressed as

6 $$\begin{aligned} X_{\text {out}} = X_{\text {out}} + X\,. \end{aligned}$$

### Vision transformer block

**Fig. 3:**
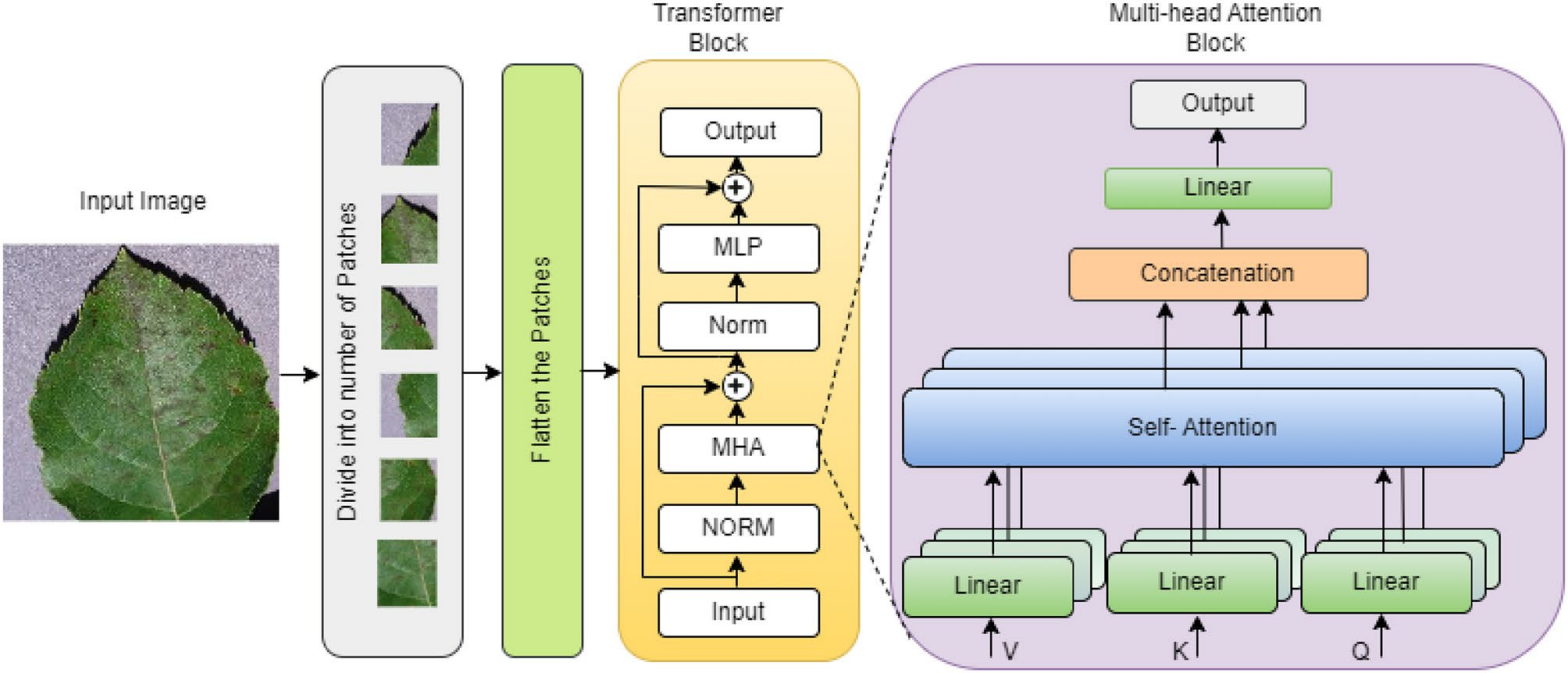
Architecture of transformer block.

With the success of transformer architecture in the natural language field, Dosovitskiy et al.^11^ used transformer architecture in image recognition task and showed that it achieves the same performance accuracy as CNN. The ViT transformer architecture consists of a multi-head attention (MHA)^40^ layer and a multi-layer perceptron (MLP) as shown in Fig. 3. Instead of dividing the image into a number of patches followed by a linear projection of patches, we pass the input image through the convolutional layer to extract the local features. The local features are divided into a number of patches, and the patches are forwarded to the transformer block. In transformer architecture, MHA and MLP are the main components, which are preceded by one normalization layer and followed by residual connection.

The self-attention mechanism calculates attention scores that represent the importance of each patch in the image. These attention scores are used to compute a weighted sum of the values (representations) of all patches, producing an attention output. Let’s denote the input to the self-attention mechanism as *X*, where *X* has dimensions $N\times d$, with *N* being the number of tokens in the sequence and *d* being the dimensionality of the token embeddings. The self-attention mechanism computes attention scores as follows:

- Query, key, and value matrices: Three matrices $W^Q,W^K$ and $W^V$, are learned parameters mapping the input *X* to query, key, and value spaces, respectively. These matrices have dimensions $d\times d$.
- Query, key, and value projections: Compute query $Q=X.W^Q$, key $K=X.W^K$ and value $V=X.W^V$
- Scaled Dot-Product Attention: Compute the scaled dot-product attention scores
  7 $$\begin{aligned} \text {Attention}(Q,K) = \texttt {softmax}\left( \frac{QK^T}{\sqrt{d}}\right) \end{aligned}$$
- Attention Output: Compute the attention output $A = \text {Attention}(Q,K).V$

The attention output *A* has dimensions $N\times d$, representing the weighted sum of values.

The ViT architecture consists of a feed forward neural network as MLP separated by a nonlinear activation function. The activation function used is Swish. The output is represented as

8 $$\begin{aligned} MLP = \texttt {Swish}(A.W + b) \end{aligned}$$

where *W* is the learnable weight matrices and *b* is the bias.

**Table 2:** Layers and parameter details of the proposed model.

Layer name	Output	Parameter
Input Layer	$256\times 256\times 3$	0
Conv2D	$128\times 128\times 16$	448
Inception A	$128\times 128\times 160$	9472
Inception B	$128\times 128\times 192$	53104
MV2	$128\times 128\times 16$	7264
MV2	$64\times 64\times 24$	1920
MV2	$64\times 64\times 24$	3216
MV2	$32\times 32\times 48$	4464
ViT	$32\times 32\times 80$	270048
MV2	$16\times 16\times 80$	28640
ViT	$16\times 16\times 96$	493472
Conv2D	$16\times 16\times 320$	31040
Global average pooling2D	320	0
Dense	4	1605
Total	–	904,693

**Fig. 4:**
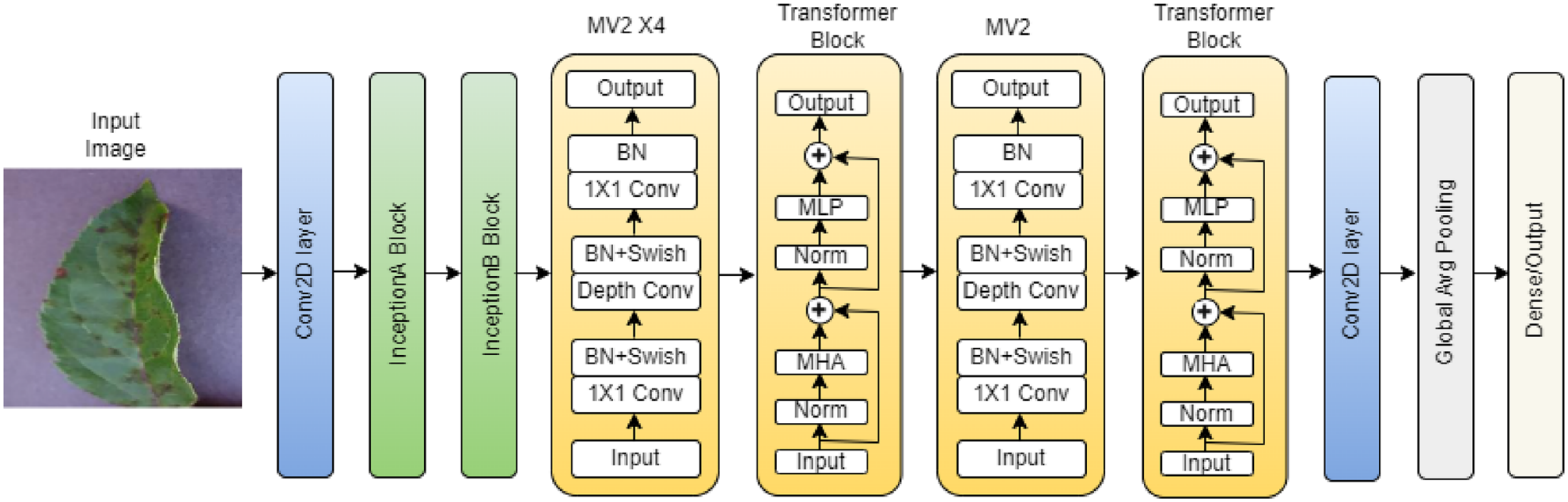
Block diagram of proposed Inception-Enhanced ViT architecture.

### Inception enhanced vision transformer architecture

The main objective of this work is to hybridize the Inception block with ViT architecture to identify the diseases in the plants. The inception block used in the architecture extracts the local features, and the ViT architecture extracts the global feature information. The inception-enhanced ViT architecture consists of a Convolution block, an InceptionA block, followed by an InceptionB block, an Inverted bottleneck Block (MV2), Vision Transformer block (ViT), and Global Average Pooling.

The architecture of Inception-enhanced ViT is shown in Fig. 4. The first layer is the input layer, which takes the RGB images of size $256\times 256\times 3$ as input. The next layer used is a convolutional layer with filter size $3\times 3$. The output generated in this layer is of size $128\times 128\times 16$. The output of the convolution layer is forwarded to the InceptionA block, followed by the InceptionB block for multilevel feature extraction. InceptionA uses filter size of $1\times 1$,$3\times 3$ depthwise convolution, and $3\times 3$ max-pooling layer as shown in Fig. 1. The InceptionB block consist of $1\times 1$,$7\times 7$ depthwise convolution, and $3\times 3$ avg-pooling layer as shown in Fig. 1. The output generated after the InceptionB block is of size $128\times 128\times 192$. The output of each layer is concatenated together and forwarded as the input of the next layer. Next, a number of MV2 blocks is used, which enhances the feature reduction as well as reduces the number of computations. The output features of the MV2 block are then divided into a number of patches of size $n\times n$ (n = 2, 4, 8) and passed through the transformer block for global feature extraction. Non-overlapping patch embedding technique is used in this work, where the input feature map is divided into a number of patches determined by the patch size. The output of the transformer block is then passed through a convolution layer and a global average pooling layer, which converts the output of the convolution layer into a 1D vector. Finally, a dense layer is used with a softmax activation function and output neuron, which is equal to the number of classes in the dataset. A brief description of the parameter used along with the output size of each layer is shown in Table 2. For an instance of 5 classes, the required parameter is 904,693, which varies with regard to the number of output classes in the dataset. The size of the required parameter is 3.72MB. The activation function used in the convolution block is ReLu, and in MV2 and transformer block is Swish.

## Experimental results and analysis

### Dataset

Five open-source publicly available dataset is used to evaluate the performance of the proposed model. The dataset used are Apple leaf image dataset^41^, rice disease dataset^34^, ibean dataset^36^, PlantVillage dataset^14^, and Cassava dataset^42^. The images in the dataset are of different categories, such as laboratory-conditioned images, field images, images with multiple leaves and images with complex backgrounds. The images used in this work is resized into $224\times 224$. The purpose of using multiple dataset is to evaluate the robustness of the proposed model.

**Fig. 5:**
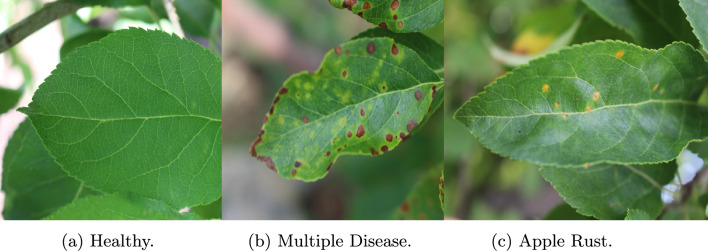
Sample images from Apple Leaf Dataset^41^.

Apple leaf image dataset^41^ is the first dataset that is used in this work, which is a freely available dataset and consists of 1820 images. Table 3 gives a brief dataset description along with number of classes. Figure 5 shows the sample images from the dataset.

**Table 3:** Apple^41^ and Rice^34^ dataset description.

Apple dataset^41^		Rice dataset^34^	
Disease name	Number of images	Disease name	Number of images
Healthy	515	Bacterial blight	1580
Multiple disease	91	Blast	1440
Scab	592	Brown spot	1600
Apple Rust	622	Tungro	1308
Total	1820	Total	5932

**Fig. 6:**
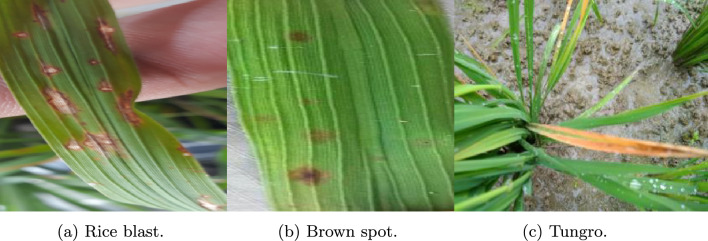
Sample images from Rice Leaf dataset^34^.

The second dataset used is the rice leaf diseases dataset^34^, which consists of 5932 images of four categories of rice diseases. The images in this dataset were captured in a real field. Figure 6 shows the sample images of the dataset, and Table 3 shows the description of the dataset.

**Fig. 7:**
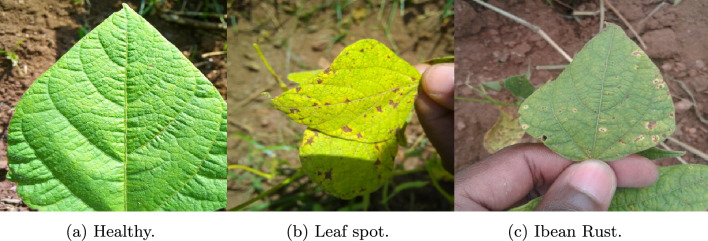
Sample images from ibean dataset^36^.

The third dataset used is ibean dataset^36^, which consists of three classes of images. The images in this dataset were captured in real-time field conditioned, and multiple leaves were present in single images. Table 4 and Fig. 7 show the dataset description and sample images from the dataset.

**Table 4:** Ibean^36^ and Cassava^42^ dataset description.

Ibean dataset^36^		Cassava dataset^42^	
Disease name	Number of images	Disease name	Number of images
Angular leaf spot	432	Cassava mosaic disease (CMD)	2658
Bean Rust	436	Cassava bacterial blight (CBB)	466
Healthy	428	Cassava green mite (CGM)	773
		Cassava brown streak disease (CBSD)	1443
		Healthy	316
Total	1296	Total	5656

**Fig. 8:**
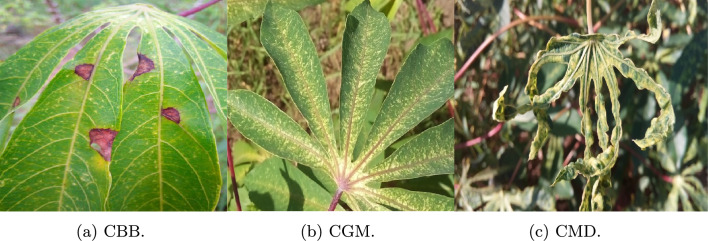
Sample images from Cassava dataset^42^.

The fourth dataset used is the cassava dataset^42^, which consists of one healthy and four disease-class images. The images in the dataset were captured with complex backgrounds. Figure 8 shows the sample images, and Table 4 shows the detailed dataset description.

**Fig. 9:**
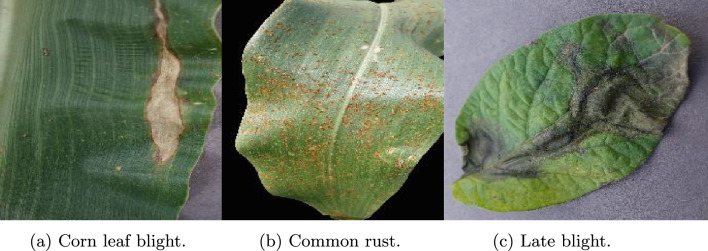
Sample images from PlantVillage dataset^14^.

The images in the fifth dataset are from the plantvillage dataset^14^. PlantVillage dataset consists of 54305 images and 38 different categories of diseases. In this work, we have considered only 7 categories of potato and corn images. Figure 9 contains the sample images, and Table 5 summarizes the detailed dataset information.

**Table 5:** PlantVillage (Corn and Potato) dataset^14^ description.

Disease name	Number of images
Corn Gray leaf spot	513
Corn Common rust	1192
Corn healthy	1162
Corn Northern Leaf Blight	985
Potato Early blight	1000
Potato Healthy	152
Potato Late blight	1000
Total	6004

### Evaluation matrics

Performance evaluation matrices determine how effectively the proposed model classifies the images. In this paper, we have used several performance matrices such as accuracy, sensitivity, specificity, precision, False Positive Rate (FPR), False Negative Rate (FNR), f1-score, and Matthews Correlation Coefficient (MCC). The proportion of accurately predicted images to all images is called accuracy.

9 $$\begin{aligned} \text {Accuracy} = \frac{\text {TP}+\text {TN}}{\text {TP}+\text {FP}+\text {TN}+\text {FN}} \end{aligned}$$

Precision is defined as the proportion of true predictions to the total number of positive predictions of the model.

10 $$\begin{aligned} \text {Precision} = \frac{\text {TP}}{\text {TP}+\text {FP}} \end{aligned}$$

Sensitivity is defined as the proportion of positive classes classified as positive to the total number of positive classes.

11 $$\begin{aligned} \text {Sensitivity} = \frac{\text {TP}}{\text {TP}+\text {FN}} \end{aligned}$$

FPR is the ratio of false predictions with negative values.

12 $$\begin{aligned} \text {FPR} = \frac{\text {FP}}{\text {FP}+\text {TN}} \end{aligned}$$

FNR is the ratio of false negative values with positive values.

13 $$\begin{aligned} \text {FNR} = \frac{\text {FN}}{\text {FN}+\text {TP}} \end{aligned}$$

F1-score is defined as the harmonic mean of precision and recall.

14 $$\begin{aligned} F1-score = 2 \,\left( \frac{\text {Precision}\times \text {Recall}}{\text {Precision}+\text {Recall}}\right) \,, \end{aligned}$$

where TP is true positive, which is defined as the correctly predicted positive along with the original class as positive. FP indicates false positive, which is defined as images supposed to be positive but predicted negatively. TN is true negative, indicating images are negative and predicted as negative. FN is false negative, indicating images are negative but predicted as positive.

### Results and discussion

**Fig. 10:**
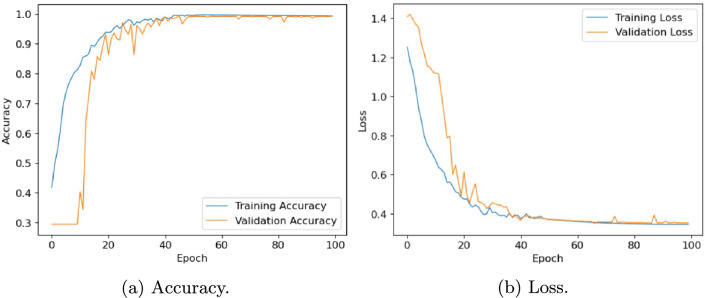
Performance on Apple Leaf dataset^41^.

**Fig. 11:**
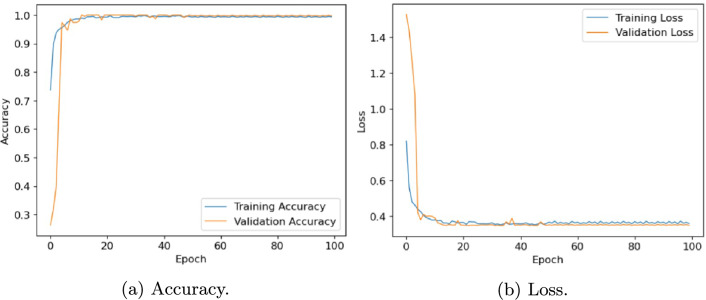
Performance on Rice dataset^34^.

**Fig. 12:**
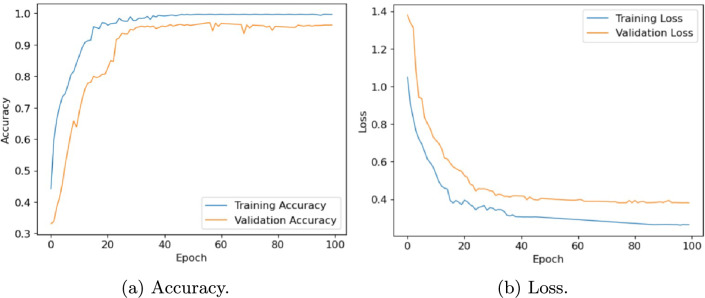
Performance on Ibean leaf dataset^36^.

**Fig. 13:**
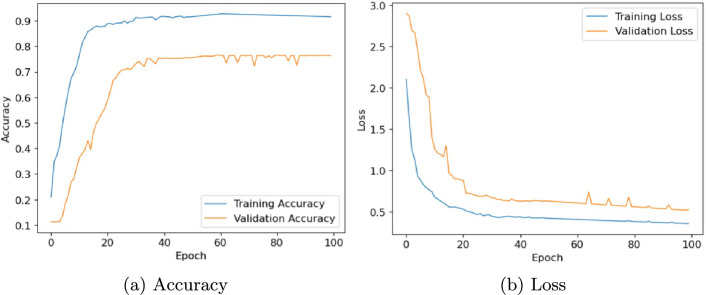
Performance on Cassava dataset^42^.

In this section, we evaluate the performance of the proposed architecture to identify diseases in plants without specifying the disease class. We consider the following five different publicly available plant disease datasets: apple leaf dataset^41^, rice leaf dataset^34^, ibean dataset^36^, cassava dataset^42^ and plantvillage dataset^14^. We set the learning rate of the proposed model as 0.001, the batch size as 32, and the training epoch as 100. Firstly, the performance of the model is evaluated in terms of accuracy and loss. Table 6 presents the training and validation accuracy, training and validation loss on five datasets with 100 epochs. Figures 10, 11, 12, 13 and, 14 shows the progression of accuracy and loss on each dataset using the Adam optimizer after 100 epochs. From the figures, we observe that the accuracy increases and the loss decreases with respect to epochs. From the accuracy and loss curve, we find that after a certain number of epochs, the accuracy and loss stabilize, thereby achieving optimum results.

**Fig. 14:**
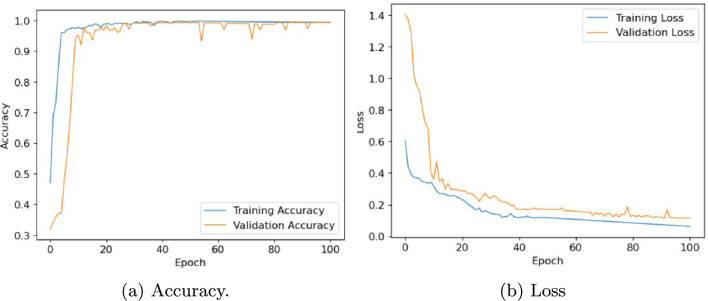
Performance on PlantVillage dataset^14^.

**Table 6:** Performance of Inception-Enhanced ViT architecture on different datasets.

Dataset	Train Acc.	Train Loss	Val Acc.	Val Loss
Apple^41^	0.9972	0.3728	0.9923	0.3732
Rice^34^	1.0000	0.3328	0.9970	0.3528
Ibean^36^	0.9965	0.3028	0.9702	0.4028
Cassava^42^	0.9247	0.4228	0.7651	0.6328
PlantVillage^14^	0.9973	0.1347	0.9941	0.1836

We have also investigated the model performance with other key performance matrices such as sensitivity, specificity, precision, FPR, FNR, f1-score, and mathews correlation coefficient (MCC). Table 7 summarizes the matrices on each dataset. Moreover, the performance of the proposed model on test images is shown in terms of the confusion matrix.

**Table 7:** Performance metrics of the proposed model on different datasets.

Dataset	Sensitivity	Specificity	Precision	FPR	FNR	F1-score	MCC
Apple^41^	0.9715	0.9971	0.9821	0.0028	0.0270	0.9767	0.9741
Rice^34^	0.9950	0.9983	0.9950	0.0017	0.0049	0.9950	0.9933
Ibean^36^	0.9690	0.9845	0.9690	0.0155	0.0309	0.9690	0.9536
Cassava^42^	0.6717	0.9317	0.7096	0.0682	0.3292	0.6855	0.6237
PlantVillage^14^	0.9945	0.9965	0.9901	0.0011	0.0054	0.9937	0.9912

**Fig. 15:**
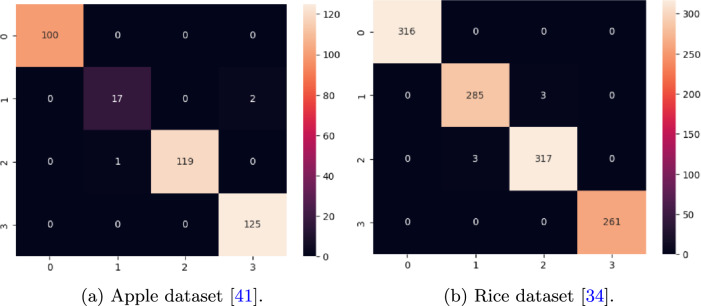
Confusion matrix on Apple and Rice dataset.

**Fig. 16:**
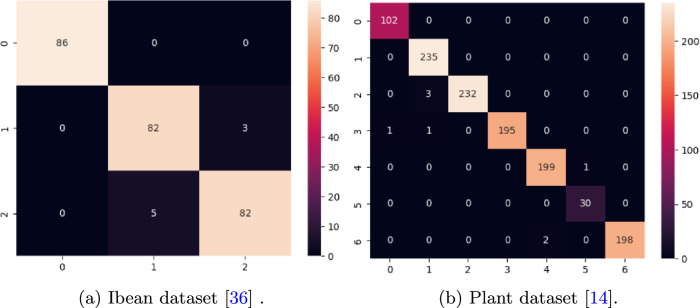
Confusion matrix on Ibean and PlantVillage dataset.

**Fig. 17:**
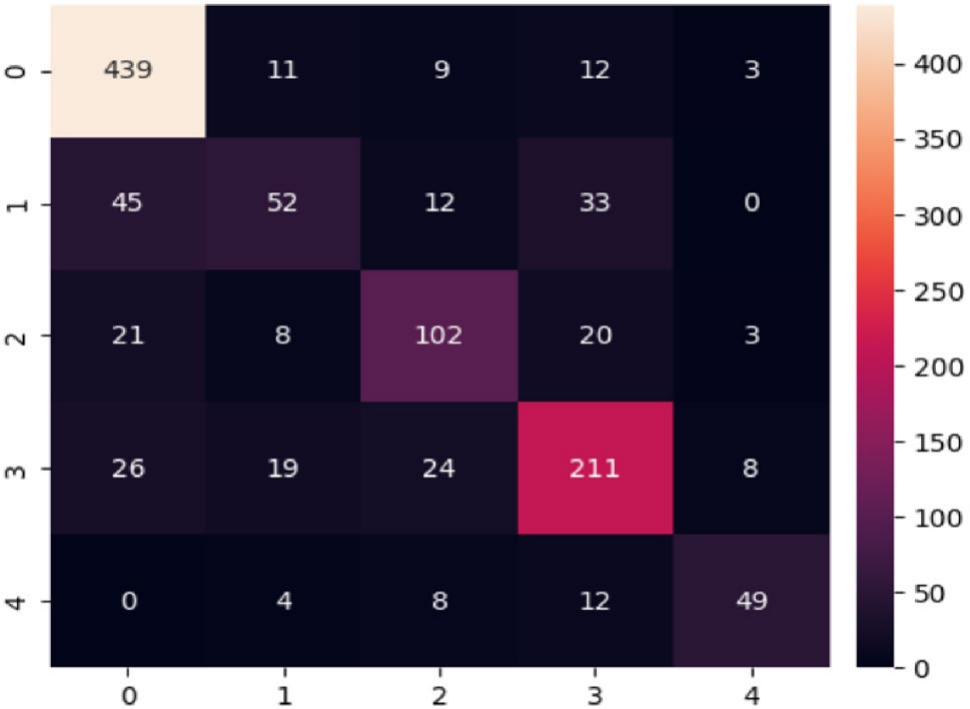
Confusion matrix of cassava dataset^42^.

The confusion matrices of apple^41^, rice^34^, ibean ^36^, cassava^42^ and plantvillage datasets^14^ are drawn and shown in Figs. 15, 16 and 17. From the confusion matrix, it is observed that the proposed model has very few false positives and false negatives in all the datasets.

#### Performance comparison with different optimizers

We evaluate and compare the performance of the proposed Inception-ViT architecture with several optimizers to find which optimizer provides the best performance. We select the following optimizers: SGD^43^, Adam^44^, RMSProp^43^, Adamax^43^, Adadelta^43^, and Ftrl^45^. From Table 9, we observe that Adam and RMSProp optimizer provide the highest performance accuracy. Moreover, we can conclude that the Adam optimizer outperforms others for all the datasets^14,34,36,41,42^.

**Table 8:** Performance comparison of the proposed model with standard DL models under similar training conditions on PlantVillage Dataset.

Models	Accuracy (%)	Precision (%)	Recall (%)	F1-score (%)	Parameter (M)	Size (MB)	FLOPs (B)
VGG16	95.65	95.54	95.72	95.62	138.4	527.79	31.04
VGG19	98.87	98.23	98.45	98.34	143.7	548.05	39.38
InceptionV3	97.27	97.31	97.28	97.29	23.9	103.61	5.72
ResNet50	97.98	97.68	97.82	97.75	25.6	97.49	8.26
EfficientNetB0	99.62	99.66	99.63	99.45	5.3	20.17	820.4
MobileNetV2	93.52	93.23	93.48	93.35	3.5	13.37	640.6
Inception Enhanced VIT	99.42	99.27	99.34	99.30	0.90	3.72	29.34

**Table 9:** Performance comparison with different optimizers.

Optimizer	Training Acc	Training loss	Validation Acc	Validation Loss
Apple dataset^41^				
SGD	0.4638	0.9856	0.3891	1.0374
RMSProp	0.9982	0.1178	0.9968	0.1251
Adam	0.9972	0.3728	0.9923	0.3732
Adamax	0.8147	0.8556	0.7023	0.8703
Adadelta	0.8238	0.5591	0.7176	0.8390
Nadam	0.8268	0.5621	0.7248	0.8232
Ftrl	0.8562	0.5394	0.7451	0.8132
Rice dataset^34^				
SGD	0.8692	0.5346	0.7318	0.6146
RMSProp	1.000	0.3393	0.9927	0.3567
Adam	1.000	0.3328	0.9970	0.3528
Adamax	0.9168	0.4285	0.8527	0.5128
Adadelta	0.8571	0.5348	0.8038	0.5793
Nadam	0.8179	0.5249	0.7748	0.6173
Ftrl	0.8027	0.5294	0.7819	0.6123
Ibean dataset^36^				
SGD	0.4152	1.0348	0.3750	1.0877
RMSProp	0.9826	0.3290	0.9294	0.4241
Adam	0.9965	0.3028	0.9702	0.4028
Adamax	0.4152	1.0348	0.3750	1.0877
Adadelta	0.4152	1.0348	0.3750	1.0877
Nadam	0.4152	1.0348	0.3750	1.0877
Ftrl	0.4152	1.0348	0.3750	1.0877
Cassava dataset^42^				
SGD	0.7682	0.6251	0.5025	0.7396
RMSProp	0.9046	0.4376	0.7381	0.6451
Adam	0.9247	0.4228	0.7651	0.6328
Adamax	0.7187	0.6429	0.4627	0.7914
Adadelta	0.7097	0.6452	0.4607	0.8014
Nadam	0.7285	0.6104	0.4821	0.7552
Ftrl	0.7047	0.6625	0.4663	0.7936
PlantVillage dataset^14^				
SGD	0.8732	0.4753	0.8271	0.5129
RMSProp	0.9952	0.1393	0.9918	0.2178
Adam	0.9973	0.1347	0.9942	0.1836
Adamax	0.9263	0.4621	0.8575	0.4726
Adadelta	0.8357	0.4961	0.7817	0.6129
Nadam	0.9136	0.3961	0.8537	0.4327
Ftrl	0.8436	0.4853	0.7971	0.5326

#### Performance with the number of patches

Moreover, to show the effectiveness of patch sizes in the proposed Inception with ViT architecture for the identification of plant diseases, we provide a comparative analysis with different patch sizes. We consider the following patch sizes: $2\times 2$, $4\times 4$, $8\times 8$, and $16\times 16$. From Table 10, we can see that the patch size has less impact on the performance. However, using patch size $8\times 8$ and $4\times 4$ provides a superior performance as compared to patch size $2\times 2$ and $16\times 16$, respectively.

**Table 10:** Performance comparison with different patch sizes.

Patch size	Training loss	Training Acc	Validation loss	Validation Acc
Apple dataset^41^				
2	0.3741	0.9912	0.3302	0.9874
4	0.3828	0.9914	0.3722	0.9914
8	0.3728	0.9972	0.3732	0.9923
16	0.3875	0.9905	0.3826	0.9841
Rice dataset^34^				
2	0.3354	0.9942	0.3671	0.9901
4	0.3249	1.0000	0.3521	0.9942
8	0.3228	1.0000	0.3528	0.9970
16	0.3485	0.9912	0.3662	0.9897
Ibean dataset^36^				
2	0.3334	0.9843	0.4264	0.9579
4	0.3157	0.9904	0.4178	0.9617
8	0.3028	0.9965	0.4028	0.9702
16	0.3394	0.9808	0.4349	0.9496
Cassava dataset^42^				
2	0.4417	0.8846	0.6579	0.7319
4	0.4259	0.9155	0.6297	0.7552
8	0.4228	0.9247	0.6328	0.7651
16	0.4491	0.8808	0.6592	0.7296
PlantVillage dataset^14^				
2	0.1507	0.9904	0.2142	0.9902
4	0.1358	0.9947	0.1857	0.9926
8	0.1347	0.9973	0.1836	0.9941
16	0.2268	0.9923	0.1926	0.9917

**Table 11:** Performance comparison of the proposed model with existing work on PlantVillage dataset^14^.

Paper Ref.	DL model used	No of class	Performance	Parameter
Mohanthy et al.^14^	Transfer learning (GoogleNet)	38	99.34	6.7M
Ferentinos et al.^15^	Transfer Learning (VGG16, Overfeat)	58	99.53	138.4M
Waheed et al.^46^	Dense CNN	3	98.06	NA
Pandey et al.^24^	DADCNN-5	38	99.93	NA
Fang et al.^47^	ResNet-50	10	95.61	25.6M
Thakur et al.^16^	VGG-ICNN	NA	99.16	6M
I Kunduracioglu^48^	EfficientNetV2_m	4	100	54.4M
I Kunduracioglu^49^	Res2Next50	10	99.85	NA
Dheeraj et al.^17^	LWDN	NA	99.37	1.5M
I Kunduracioglu et al.^50^	CNN with ViT	4	100	NA
Proposed	Inception-Enhanced ViT	7	99.41	0.90M

### Comparison with state-of-art deep learning models

In order to verify the robustness of the proposed model, we have compared the performance of the proposed model with several state-of-the-art deep learning architectures. The performance comparison of the proposed model with different deep learning models has been summarized in Table 8 after 100 epochs. From Table 8, it is observed that the proposed Inception-enhanced Vision Transformer model outperforms the state-of-the-art deep learning architectures. Table 8 also compares the parameters required of the deep learning models, and it shows that the proposed deep learning model uses fewer parameters. Table 8 shows the size and Floating Point Operations per Second (FLOPs) required in each model, and it shows that the proposed model require fewer FLOPs as compared to standard DL models. The performance of the proposed model is also compared with the other deep learning models in Table 11. From Table 11, it is noted that the proposed model can successfully classify diseases in plants with higher performance accuracy as compared to the existing works. Hence, it is worthwhile to note that the proposed Inception-enhanced Vision Transformer outperforms state-of-the-art deep learning models.

## Conclusion

In this paper, we propose an inception-enhanced Vision Transformer architecture to identify diseases in plant leaves. The fusion of inception in ViT architecture has the benefit of having both local and global feature extraction, which increases the performance of the model. In fact, the ViT blocks in the proposed model accelerate the training, and the attention model in ViT focuses on the meaningful regions in the input image. An investigation is performed with different patch sizes to achieve the optimal architecture. The results reveal that Inception-enhanced ViT with 4 patch size gives the best performance accuracy. The proposed inception-enhanced ViT architecture is experimented on five different datasets, which are unbalanced datasets, images in the dataset with complex backgrounds, and images with multiple leaves. The experimental result shows that the model performance achieves impressive results with an accuracy rate of 99.23%, 99.70%, 97.02%, 76.51%, and 99.41% on apple^41^, rice^34^, ibean^36^, cassava^42^, and plantvillage datasets^14^, respectively. The performance in the cassava dataset^42^ is lower than that of the other dataset. This is because the dataset is unbalanced and the presence of multiple leaves in a single image. Moreover, the images in the dataset are highly correlated with each other. In^51^, the authors recorded an accuracy rate of 52.87% and 46.24% using plain and deep residual convolutional neural networks in an imbalanced cassava dataset^42^. In comparison with this, our proposed model achieved a much higher performance accuracy rate in the imbalanced dataset. The number of parameters required in the proposed model is much less and can be easily deployable in small devices like smartphones. Furthermore, the likelihood of human error and disease transmission is decreased by quick and automatic identification. Moreover, the presence of agro experts in remote areas is very few; the proposed inception-enhanced Vision Transformer model provides significant benefits to the farmers to reduce crop yield loss and identify the diseases in plants in an easy manner. The future direction of this work can be extended to a real-time AI model by integrating IoT-enabled smart cameras for continuous, automated disease detection in farms. Additionally, federated learning can be employed, allowing the model to train across distributed farm data without sharing raw images.

## Data Availability

The datasets generated and/or analysed during the current study are available in Kaggle repository at: https://www.kaggle.com/datasets/piantic/plantpathology-apple-datasethttps://www.kaggle.com/datasets/therealoise/bean-disease-datasethttps://www.kaggle.com/datasets/sinadunk23/behzad-safari-jalalhttps://www.kaggle.com/datasets/mohitsingh1804/plantvillage, https://www.kaggle.com/datasets/nirmalsankalana/cassava-leaf-disease-classification.
